# Supplementary material for: Identification of PPARG as key gene to link coronary atherosclerosis disease and rheumatoid arthritis via microarray data analysis
Source: PLoS One. 2024 Apr 4;19(4):e0300022. doi: 10.1371/journal.pone.0300022 (PMC10994321; doi:10.1371/journal.pone.0300022)
Supplement: S1 Raw data — (DOCX) [file pone.0300022.s001.docx]

The raw datasets for this study can be found in the file named “”supplementary raw data” wih link https://www.jianguoyun.com/p/DUSj-IYQqdzxCRj2uLUFIAA
