# Supplementary material for: Identification of PPARG as key gene to link coronary atherosclerosis disease and rheumatoid arthritis via microarray data analysis
Source: PLoS One. 2024 Apr 4;19(4):e0300022. doi: 10.1371/journal.pone.0300022 (PMC10994321; doi:10.1371/journal.pone.0300022)
Supplement: Attachment — Submitted filename: comment.docx [file pone.0300022.s002.docx]

This study identifies 737 DEGs associated with diseased groups in CAD datasets and 2008 DEGs associated with diseased groups in RA datasets. The author observed that TUBB2A, FKBP5, PPARG were significantly associated with RA and CAD. Major revisions are requested for this paper to be accepted.

Major comments:

1. Although the authors made some efforts attempting to integrate several existing gene expression and datasets via GO and GSEA enrichment that they chose for over-represented functional gene sets, validation experiments that could support the authors' conclusion are missing, sufficient new/validated information is lacking.

2. This study need more work in order to identify more datasets or use data from authors' laboratory to show replication of the results.

3. The authors need to use a PCA and a regression SVM (R-SVM) to obtain a global view and evaluate the performance of DEGs in classifying control conditions versus CAD and RA conditions.

4. Lines 75 to 85 are not related to the introduction.

5. Most of the Figures are not clear eg, Fig 2 (A,B), Fig 4 (A,D,E), Fig 5 (A,B,C,D,F) , Fig 6 (E) and Fig 7 (A,B,C,D,E). The authors provide clearer Figures.
