# Supplementary material for: Identification of PPARG as key gene to link coronary atherosclerosis disease and rheumatoid arthritis via microarray data analysis
Source: PLoS One. 2024 Apr 4;19(4):e0300022. doi: 10.1371/journal.pone.0300022 (PMC10994321; doi:10.1371/journal.pone.0300022)
Supplement: Attachment — Submitted filename: Response to Reviewers.docx [file pone.0300022.s003.docx]

This study identifies 737 DEGs associated with diseased groups in CAD datasets and 2008 DEGs associated with diseased groups in RA datasets. The author observed that TUBB2A, FKBP5, PPARG were significantly associated with RA and CAD. Major revisions are requested for this paper to be accepted.

**Major comments:**

1. **Although the authors made some efforts attempting to integrate several existing gene expression and datasets via GO and GSEA enrichment that they chose for over-represented functional gene sets, validation experiments that could support the authors' conclusion are missing, sufficient new/validated information is lacking.**

Dear Reviewer,

Thank you for your valuable feedback.

In addition to bioinformatics analysis, our immunohistochemistry data validate the differential expression and localization of PPARG on synovial tissue and arterial wall samples from RA and CAD patients. The lack of validation in vivo and in vitro experiments in indeed the limitation of our study. Unfortunately, due to time constraints, reagent availability, and current financial limitations, we were unable to perform the validation experiments as part of this study.However, we want to assure you that the validation experiments are an integral part of our future research plan.

Moreover, we speculated that the identified hub genes PPARG can gap RA and CAD through macrophages mediated Fc-gamma receptor-mediated endocytosis based on the following reference

1 Chawla A, Barak Y, Nagy L, Liao D, Tontonoz P, Evans RM. PPAR-gamma dependent and independent effects on macrophage-gene expression in lipid metabolism and inflammation. Nat Med. 2001 Nov;7(1):48-52. doi: 10.1038/83336. PMID: 11135614. Which support PPARG function in lipid metabolism induced inflammation, including cornary artery disease.

2 Toobian D, Ghosh P, Katkar GD. Parsing the Role of PPARs in Macrophage Processes. Front Immunol. 2021 Dec 22;12:783780. doi: 10.3389/fimmu.2021.783780. PMID: 35003101; PMCID: PMC8727354. Which provide evidence to support that PPARG has been implicated in regulating macrophage polarization, phagocytosis, inflammatory cytokine production, and lipid metabolism .

3 Brosig L, Hong J, Wallis BB, Giacomini JC, Assimes TL, Goronzy JJ, Weyand CM. Hypermetabolic macrophages in rheumatoid arthritis and coronary artery disease due to glycogen synthase kinase 3b inactivation. Ann Rheum Dis. 2018 Jul;77(7):1053-1062. doi: 10.1136/annrheumdis-2017-212647. Epub 2018 Feb 3. PMID: 29431119. Which support that the macrophages implicated in the pathogenesis of both RA and CAD

2. **This study need more work in order to identify more datasets or use data from authors' laboratory to show replication of the results.**

Dear Reviewer,

Thank you for your valuable feedback.

In this study, we have carried out several verification steps. Firstly, 25 DEGs obtained from the intersection of DEGs in RA (GSE5523+GSE55457) and CAD (GSE42148) datasets and WGCNA were validated in validation datasets(RA GSE12021 and CAD GSE20680), referenced to (Fig.2)Additionally, we performed PCA and SVM analysis ( Fig.3 ) and ROC analysis to identify diagnostic discrimination ability in disease group and normal group of the 25 core genes and the three hub genes on both training and validation datasets(Fig.5) . Then qRT-PCR was conducted to validate the mRNA expression level of three hub genes in RA and CAD disease compared with their matched normal tissues. Finally, we conducted immunohistochemistry experiments to confirm the expression and localization of the core gene PPARG in synovial tissue and arterial wall macrophages from patients with chronic synovitis and coronary artery disease to support our bioinformatic study from clinical aspects (Fig.9)

We acknowledge the importance of replicating our findings to enhance the robustness of our study. However, we carefully considered various factors in our analysis, including sample size matching, sample types, result integration of the article logic, which led us to prioritize the mentioned datasets. Furthermore, the experimental validations will be supplemented in future studies and grant applications.

3. **The authors need to use a PCA and a regression SVM (R-SVM) to obtain a global view and evaluate the performance of DEGs in classifying control conditions versus CAD and RA conditions.**

Dear Reviewer,

Thank you for your valuable feedback and suggestions.

We obtained the 25 core genes from the intersection of DEGs in RA (GSE5523+GSE55457) and CAD (GSE42148) datasets and WGCNA. To validate the discriminatory ability of 25 core genes in distinguishing disease group from normal group in both RA and CAD, the expression matrix was extracted from the training and validation datasets of RA and CAD . PCA analysis was performed on the new datasets using R packages FactoMineR and factoextra . SVM analysis was then conducted using functions from the R package e1071. Finally, the classification performance of the 25 core genes was visualized by ROC curves using pROC package.Fig.5 showed the 25 core genes distinguished the disease group from the normal group in both the RA and CAD training and validation datasets with AUCs above 0.8.

4. **Lines 75 to 85 are not related to the introduction.**

Dear Reviewer,

Thank you for your valuable feedback.

We have deleted the content of line75 to 85 in the introduction and revised as follows:

Potential targets and regulatory biological pathways were identified in RA and CAD datasets respectively using microarray bioinformatics technology in recent years, however, there is a dearth of studies that investigate the macrophages related inflammatory processes between RA and CAD. This research aims to fill this gap by employing GSEA analysis to identify common signaling pathways on RA and CAD datasets.WGCNA and ssGSEA will identify module genes linked to Fc-gamma receptor phagocytosis. Three hub genes will be extracted using STRING and Cytoscape, then assessing their predictive ability in both diseases through ROC analysis. GeneMANIA and the Cibersort algorithm will provide insights into immune infiltration and transcription factors associated with the hub genes. Notably, the involvement of PPARG and macrophages in RA and CAD will be highlighted. Understanding these processes could offer new insights into the pathogenesis shared by both diseases and potentially aid in the development of targeted therapies.

5. **Most of the Figures are not clear eg, Fig 2 (A,B), Fig 4 (A,D,E), Fig 5 (A,B,C,D,F) , Fig 6 (E) and Fig 7 (A,B,C,D,E). The authors provide clearer Figures.**

Thank you for your feedback on the clarity of our figures. We appreciate your attention to detail and would like to clarify the issue you mentioned.

We have compared the raw figures in TTF format and the figures of PDF versions, the latter indeed have lower resolution and pixel quality compared to the TTF versions. I think the review can try to accesse the raw figures in TTF format using the provided link in the top right corner of the PDF figures as marked in the following attached figures.We apologize for any inconvenience caused and appreciate your understanding.

If you have any further questions or concerns, please do not hesitate to let us know. We appreciate your time and effort in reviewing our work.

Pdf	figure

TIFF figure

**Editor Comments**

1. **Fig 1. Need more explanation in legend.**

Thank you for your feedback on Fig 1 legend. We have revised as follows: ＂**Fig 1. Data analysis flowchart.** Schematic flowchart of data aquirement, processing ,analysis and validation.＂

2. **The text need to be improved as English Grammar and scientific frame.**

Thank you for your suggestions. We have made revise by the editors and the proofreading certificate was uploaded in the supplementary materials.

3. **Space and “,” should be checked in the whole text.**

Thank you for your remind. We have checked the whole text to correct this mistake.

4. **Some DE genes should be validated using qRT-PCR or other techniques.**

Thank you for your suggestions. We have performed qRT-PCR to validate TUBB2A, PPARG and FKBP5 mRNA expression in RA and CAD tissues and their mathced adjacent normal tissues.The results was demonstrated and in the results “GO and KEGG pathway analysis of 25 susceptible genes in RA and CAD”and showed in Fig.6G-H

5. **Quality of figures should be improved and the text and details should be clear.**

Thank you for your suggestions. We have assembled our figures with adobe illustration software and upload the figure again. If there is something unsatisfactory, please don’t hesitate to contact us.

Best wishes!

Yours sincerely!
