# Supplementary material for: Identification of PPARG as key gene to link coronary atherosclerosis disease and rheumatoid arthritis via microarray data analysis
Source: PLoS One. 2024 Apr 4;19(4):e0300022. doi: 10.1371/journal.pone.0300022 (PMC10994321; doi:10.1371/journal.pone.0300022)
Supplement: Attachment — Submitted filename: Comment 2.docx [file pone.0300022.s004.docx]

The authors have responded to previous comments, but revisions are needed for a possible final acceptance.

1. Briefly refer to the used data in the abstract of the method section.

2. The reference gene should be specified in the real-time PCR section.

3. What method has been used to analyze real-time PCR data?
