# Supplementary material for: Identification of PPARG as key gene to link coronary atherosclerosis disease and rheumatoid arthritis via microarray data analysis
Source: PLoS One. 2024 Apr 4;19(4):e0300022. doi: 10.1371/journal.pone.0300022 (PMC10994321; doi:10.1371/journal.pone.0300022)
Supplement: Attachment — Submitted filename: comments.docx [file pone.0300022.s005.docx]

The study is very interesting, as well as, the manuscript is improved very well, in my opinion, you can accept it. However, it is recommended to edit the following comments.

1. Generally, the pronouns (such as I, we, us) are avoided to be written in a technical research article. In the regard, try to reframe the text, e.g., line 132, 173, 216, 277, 281, 303, etc.
2. There is a blank page between the 154 and 156 lines. Please edit this error.
3. The diagrams quality is quite low. It is required to include high-resolution pictures for further comments.
4. Please review the numbers of figures. I think you mean “Figure 6” in line: 278, 282, 287, etc. and after the above-mentioned lines.
5. Please double check for spelling and English grammar errors (e.g., Space and “,”, and integral and correct time tense for whole document) in the manuscript.
6. In line 317, please clearly indicate the corresponding figure.
7. It seems lines 382-394 are redundant in the current position. It is recommended to insert the “Conclusion” section to state your total conclusion of the findings.
8. It is recommended to use an integral format of abbreviations and full form of terms, e.g. rheumatoid arthritis in line 437.
9. Please double-check the author’s citation format in lines 435, 458, etc.
10. Please double check the formats of all references.

Best wishes
