# Supplementary material for: Identification of PPARG as key gene to link coronary atherosclerosis disease and rheumatoid arthritis via microarray data analysis
Source: PLoS One. 2024 Apr 4;19(4):e0300022. doi: 10.1371/journal.pone.0300022 (PMC10994321; doi:10.1371/journal.pone.0300022)
Supplement: Attachment — Submitted filename: Rebutt letter.docx [file pone.0300022.s007.docx]

Response for comments

Reviewer #1: The study is very interesting, as well as, the manuscript is improved very well, in my opinion, you can accept it. However, it is recommended to edit the following comments.

1. Briefly refer to the used data in the abstract of the method section

**Response:**Thank you for your comment. I have added the names of the datasets in the revised abstract of the method section as follows“The study utilized five raw datasets: GSE55235 , GSE55457 , GSE12021 for RA patients, and GSE42148 and GSE20680 for CAD patients in line 24-25”

2. The reference genes should be specified in the real-time PCR section

**Response:** Thank you for your comment. I have specified the reference genes as follows”GAPDH was used as reference gene” in line 165

3. What method has been used to analyze real-time PCR data?

**Response:** Thank you for your comment. I have mentioned the method usde to analyze real-time PCR data as follows”Gene expression was calculated according to the 2-ΔΔCT method .” in line 165-166.

Reviewer #3

The study is very interesting, as well as, the manuscript is improved very well, in my opinion, you can accept it. However, it is recommended to edit the following comments.

1. Generally, the pronouns (such as I, we, us) are avoided to be written in a technical research article. In the regard, try to reframe the text, e.g., line 132, 173, 216, 277, 281, 303, etc.

**Response:** Thank you for your comments. We have made revisions to the text accordingly. And the “revised manuscript with track changes” has been submitted for your review.

2. There is a blank page between the 154 and 156 lines. Please edit this error.

**Response:** Thank you for pointing out the error. We apologize for the oversight. We have now removed the blank page between lines 154 and 156 as shown in the “revised manuscript with track changes”.

3. The diagrams quality is quite low. It is required to include high-resolution pictures for further comments.

**Response:** Thank you for your comments. We have already recombined the raw PDF images using Adobe Illustrator to generate vector graphics, exported them in TTF format with a PPI of 300dpi, then checked and adjusted in the PACE system before re-uploaded them in the submitting system, The original images remain clear even when enlarged by 150%, which can be obtained from link on the the right corner of the pdf files. I hope it will be OK. I think figures in the pdf files may be compressed too much.

Figures downloaded

4. Please review the numbers of figures. I think you mean “Figure 6” in line: 278, 282, 287, etc. and after the above-mentioned lines.

**Response:**Thank you for your comment. We have now reviewed the number of figures in the manuscript and made the necessary changes in lines 278, 282, 287, and other relevant lines, . The revised manuscript with track changes has been submitted for your review.

5. Please double check for spelling and English grammar errors (e.g., Space and “,”, and integral and correct time tense for whole document) in the manuscript.

**Response:** Thank you for your comment. We have now thoroughly reviewed the manuscript for spelling and English grammar errors. This includes checking for spaces, commas and the correct tense .The revised manuscript with track changes has been submitted for your review.

6. In line 317, please clearly indicate the corresponding figure.

**Response:** Thank you for bringing this to our attention. We have revised the manuscript according to your comment and clearly indicated the corresponding figure in line 317. We have made these changes in the revised manuscript with track changes.

7. It seems lines 382-394 are redundant in the current position. It is recommended to insert the “Conclusion” section to state your total conclusion of the findings.

**Response:** Thank you for your valuable suggestions. We have revised the manuscript accordingly. The redundant sections from lines 382-394 have been removed, and a "Conclusion" section has been inserted to provide a comprehensive summary of our findings. The revised manuscript with track changes has been submitted for your review.

8. It is recommended to use an integral format of abbreviations and full form of terms, e.g. rheumatoid arthritis in line 437.

**Response:** Thank you for your comment. We have made the necessary changes in the revised manuscript with track changes, Except for the first occurrence in the abstract and the introduction, where 'rheumatoid arthritis' is used in full form, the abbreviation 'RA' is used throughout the rest of the manuscript. Please review the revised manuscript for detail.

9. Please double-check the author’s citation format in lines 435, 458, etc.

**Response:** Thank you for your comment. I am not entirely sure about the issue with the citation format in lines 435 and 458, as I have checked and found that they correspond with the numbering of the references listed below. The citation format is consistent with the above ones as well and the published article in “plos one”. Could you please provide more specific details about the problem? Thank you for your patience.

10. Please double check the formats of all references.

**Response:** Thank you for your comment. I have checked all the references and replaced reference 7 from Microsoft Azure platform with other reference. Some references have been supplemented with published articles from Plos One, including volumes (issues) and DOI links following the published article in Plos One.Please review the revised manuscript for detail.

**Journal requirements**

Please review your reference list to ensure that it is complete and correct. If you have cited papers that have been retracted, please include the rationale for doing so in the manuscript text, or remove these references and replace them with relevant current references. Any changes to the reference list should be mentioned in the rebuttal letter that accompanies your revised manuscript. If you need to cite a retracted article, indicate the article’s retracted status in the References list and also include a citation and full reference for the retraction notice.

**Response:** Thank you for your comment. I have checked all the references and replaced reference 7 from Microsoft Azure platform with other reference. Some references have been supplemented with published articles from Plos One, including volumes (issues) and DOI links following the published article in Plos One.Please review the revised manuscript for detail. None of the reference was restracted by now after throughly checked in the pubmed.
